# Promising immunotherapeutic targets for treating candidiasis

**DOI:** 10.3389/fcimb.2024.1339501

**Published:** 2024-02-09

**Authors:** Zhe Feng, Hui Lu, Yuanying Jiang

**Affiliations:** Department of Pharmacy, Shanghai Tenth People’s Hospital, School of Medicine, Tongji University, Shanghai, China

**Keywords:** candidiasis, immunotherapy, antibody, vaccine, *Candida albicans*

## Abstract

In the last twenty years, there has been a significant increase in invasive fungal infections, which has corresponded with the expanding population of individuals with compromised immune systems. As a result, the mortality rate linked to these infections remains unacceptably high. The currently available antifungal drugs, such as azoles, polyenes, and echinocandins, face limitations in terms of their diversity, the escalating resistance of fungi and the occurrence of significant adverse effects. Consequently, there is an urgent need to develop new antifungal medications. Vaccines and antibodies present a promising avenue for addressing fungal infections due to their targeted antifungal properties and ability to modulate the immune response. This review investigates the structure and function of cell wall proteins, secreted proteins, and functional proteins within *C. albicans*. Furthermore, it seeks to analyze the current advancements and challenges in macromolecular drugs to identify new targets for the effective management of candidiasis.

## 1 Introduction

Over the last few decades, candidiasis has gradually increased morbidity and mortality rates, primarily caused by *Candida albicans* (Lu et al., 2023b). Typically, *C. albicans* exist as non-pathogenic symbiotic fungi in healthy individuals, predominantly colonizing the surface of human mucosa, including the skin, oral and pharyngeal regions, gastrointestinal tract, and urogenital tract (Williams et al., 2013; Lu et al., 2023a). When the human immune system is compromised, *C. albicans* can rapidly transition from non-pathogenic to pathogenic fungi, resulting in superficial or deep candidiasis, including thrush and candidemia. Invasive candidiasis has a global annual impact on over 1,565,000 individuals, resulting in approximately 1,000,000 fatalities (Denning, 2024). Hence, candidiasis emerges as a worldwide concern, necessitating significant consideration due to its impact on human well-being.

Currently, the clinical practice utilizes only three categories of small-molecule antifungal medications: polyenes, azoles, and echinocandins (Robbins et al., 2016). However, these antifungal agents are associated with certain limitations, which impede their clinical utility for treating candidiasis (Cowen et al., 2014). Polyenes can induce significant adverse effects as a result of the structural resemblance between the intended target, ergosterol, and cholesterol, a sterol found in mammalian cell membranes (Robbins et al., 2017). Despite their potent fungicidal properties and commendable safety profile, echinocandins face limitations in terms of their narrow antifungal range, requirement for intravenous administration, and high cost, which pose challenges to their clinical application (Perlin, 2011; Pappas et al., 2016). Azoles, on the other hand, exhibit low toxicity levels and a broad spectrum of antifungal activity. However, their fungistatic effects in certain species have led to the emergence of azole-resistant isolates (Robbins et al., 2017; Nishimoto et al., 2020). Significantly, the effectiveness of these drugs in combating fungal infections is notably reduced in patients with compromised immune function when compared to individuals with an intact immune system (Logan et al., 2020). Consequently, despite advancements in antifungal therapy, patients with invasive candidiasis experience significant morbidity and mortality. Hence, the urgency for exploring and implementing alternative therapeutic strategies to manage invasive candidiasis caused by *Candida* species effectively cannot be overstated.

The human immune system responds to candidiasis through two distinct mechanisms: innate and adaptive immunities (Lu et al., 2023b). The innate immune system primarily consists of physical barriers, such as mucosal epithelial surfaces in the skin, mouth, upper respiratory tract, gastrointestinal tract, and urogenital tract (Nami et al., 2019). Upon fungal infection, pathogen-associated molecular patterns (PAMP) that are currently present in *C. albicans* are recognized by pattern recognition receptors (PRRs), such as Integrin, Lectin, and Toll-like receptors (TLRs), which trigger the production of inflammatory factors and the recruitment and activation of phagocytes to fight the infection (Vonk et al., 2006). Moreover, the fungus that has infiltrated is taken up and subjected to phagocytosis by resident macrophages, dendritic cells (DCs), and polymorphonuclear neutrophils (PMN). Additionally, macrophages and PMN release antimicrobial peptides (AMP), inflammatory cytokines, and chemokines (Netea et al., 2004; Martinez et al., 2009). Macrophages can phagocytose and destroy *Candida* yeast cells, reducing the fungal load as soon as an infection occurs (Jones-Carson et al., 1997). Monocytes contacted *Candida* early in the infection and were more effective than DCs or macrophages in destroying *C. albicans* (Netea et al., 2004). Monocyte-deficient mice are more susceptible to *C. albicans* infections, and monocytes exposed to *C. albicans* release tumor necrosis factor-α (TNF-α), which protects the host from invasive candidiasis (Netea et al., 1999; Ngo et al., 2014). Neutrophils can also release neutrophil extracellular traps (NETs) that capture *C. albicans* (Matejuk et al., 2010). Since neutrophil activation is necessary to eradicate *Candida* species and neutropenia is a significant risk factor for invasive fungal infections, neutrophils are vital in the host’s defense against candidiasis (Newman and Holly, 2001). Neutrophils have been shown to have a role in *C. albicans* death, and they are the only immune cells capable of successfully preventing the transition of *C. albicans* blastopores to hyphae (Lionakis and Netea, 2013; Lionakis, 2014). Natural killer (NK) cells can directly kill *C. albicans* by secreting cytotoxic molecules (Voigt et al., 2014). In addition, the complement system is a component of innate immunity and is critical in the host’s fight against *C. albicans* (Harpf et al., 2020). Along with its capacity to promote phagocytosis by opsonization, the complement system’s activation produces anaphylatoxins (C3a, C4a, and C5a) responsible for several pleiotropic effects (Brown et al., 2012). As DCs can phagocytose and present antigens, they play a significant part in the host’s defense against invasive candidiasis. This links innate and cell-mediated antifungal immunity (Newman and Holly, 2001; Romagnoli et al., 2004). Therefore, in addition to playing a crucial role in initiating early defense against fungal infections, the innate immune system also triggers various responses promoted by the adaptive immune system through DCs (Medici and Del Poeta, 2015). Cell-mediated immunity and humoral immunity are two types of adaptive immunity essential for removing invasive pathogenic fungi and creating powerful immune defenses. The host’s main defense against *Candida* species is cell-mediated immunity, and CD4^+^ T helper cells and CD8^+^ cytotoxic T-cells are the main players in preventing invasive candidiasis (Medici and Del Poeta, 2015). It has long been believed that the CD4^+^T cell immune response protects against *Candida* infection (Conti and Gaffen, 2010). T cells with CD4^+^ function participate in the antifungal immune response through Th1, Th2, and Th17 immunity (Richardson and Moyes, 2015). On the other hand, the direct cytotoxic activity and cytokine release of CD8^+^ T-cells are known to mediate resistance to systemic fungal infections (Ashman et al., 1999). Cytokine secretion (mainly interferon-gamma (IFN-γ) and TNF-α) has been demonstrated as one of the main effector mechanisms through which CD8^+^ T-cells can restrict fungal infection (Gozalbo and Gil, 2009). Antibodies primarily facilitate humoral immunity, which initiates complement activation, opsonization, and antibody-dependent cellular cytotoxicity (Casadevall et al., 2002). As a result, these antibodies neutralize toxins, hinder the adherence of pathogenic fungi to host cells, impede biofilm formation, inhibit germ tube formation, and restrict fungal load (Pathakumari et al., 2020). When the equilibrium between the host immune response and fungus is disrupted, as seen in cancer chemotherapy and immunotherapy, the heightened utilization of organ transplantation and immunosuppressive agents, along with the extensive application of broad-spectrum antibiotics, will lead to an increased pathogenic behavior of *C. albicans* in immunocompromised patients, resulting in the dissemination of *C. albicans* organs (Cassone and Cauda, 2012; Del Poeta and Casadevall, 2012).

**Figure 1:**
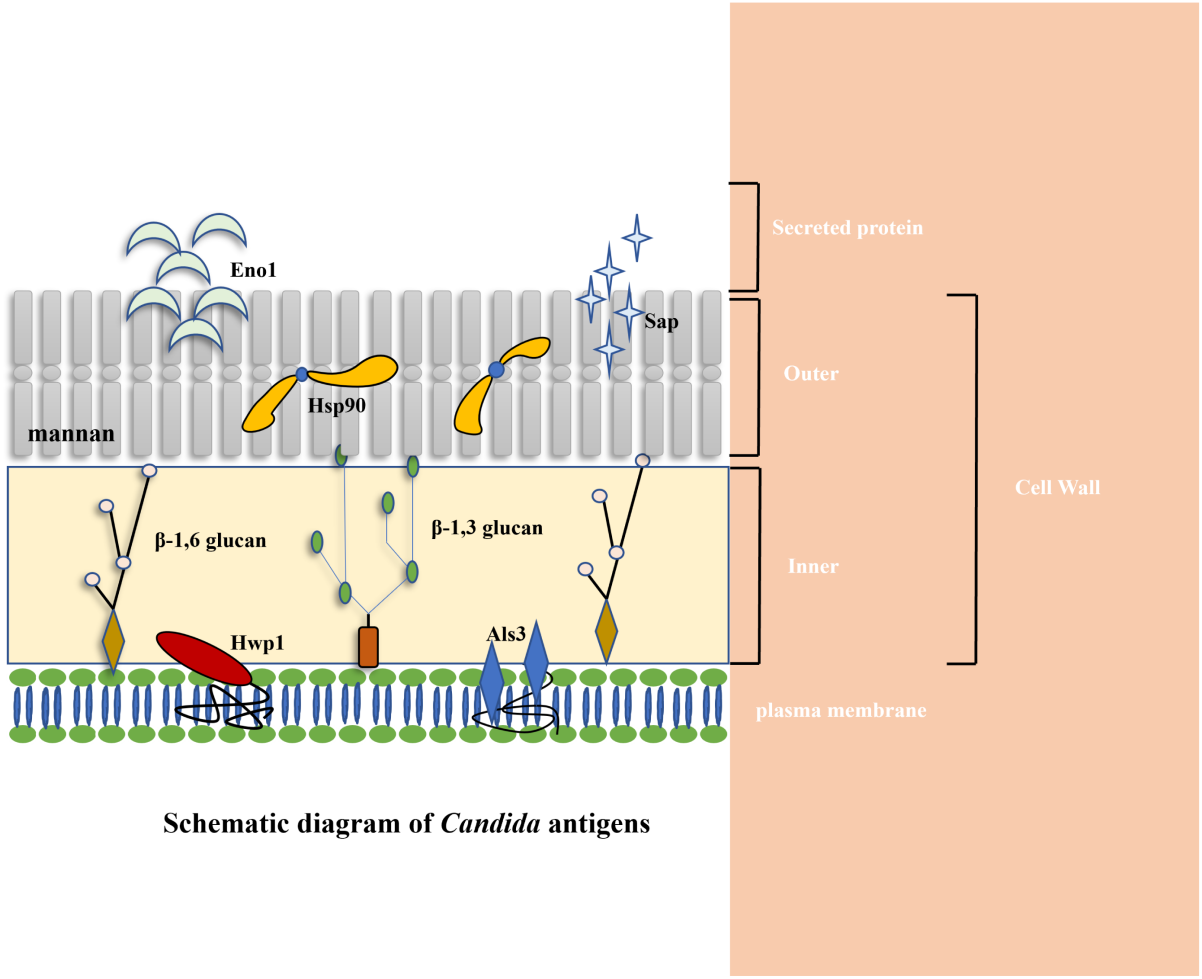
Schematic diagram of *C. albicans* antigens. Cytoplasmic location of Hsp90 and Eno1 is indicated along with various cell surface antigens (Als3 and Hwp1). Cell surface is to indicate secreted (Eno1, Sap2), and cell wall associated antigens (Hwp1, Sap2, Als3, Eno1, Hsp90) (Matthews and Burnie, 1988; Matthews and Burnie, 1989; Staab et al., 1996; Felk et al., 2002; Naglik et al., 2003; Burnie et al., 2006; Hoyer et al., 2008; Calugi et al., 2013; Silva et al., 2014; Chatterjee and Tatu, 2017; Shukla et al., 2021).

Immunotherapeutic approaches exhibit potential as a novel strategy for treating candidiasis, owing to the significant involvement of the human immune system in managing this condition. Immunotherapies encompass therapeutic approaches aimed at targeting and impacting the immune system of the body, thereby enhancing the host’s ability to combat infections (Qadri et al., 2023). These methodologies encompass various strategies, such as augmenting the population of phagocytes, activating innate defense pathways in phagocytes and DCs, and stimulating antigen-specific immunity through means like vaccines and monoclonal antibodies. It is crucial to customize immunotherapy to suit the specific immunocompromised conditions of individuals (Segal et al., 2006). In contrast to prior literature that primarily focused on elucidating the mechanisms underlying fungal infection and the immune system’s reaction to fungi (Segal et al., 2006; Armstrong-James and Harrison, 2012; Nami et al., 2019), the present review provides a comprehensive analysis of the diverse proteins present on the surface of *C. albicans*, which are pivotal in the development of candidiasis (**Table 1**) (**Figure 1**). Moreover, investigations into the efficacy of antibodies and vaccines targeting these proteins have revealed promising therapeutic outcomes. This review critically examines the structural characteristics and functional roles of these proteins in candidiasis, as well as explores the associated antibodies and vaccines. This review proposes that employing immunotherapeutic approaches holds promise as an innovative treatment of candidiasis strategy.

**Table 1:** List of vaccines and monoclonal antibodies against *C.albicans* antigens.

Target (*C.albican*s antigens)	Description (vaccine/antibodies)	Clinical trial stage	Reference
Als3	rAls3p-N(vaccine)	Not determined	(Spellberg et al., 2006; Lin et al., 2008; Lin et al., 2009)
	NDV-3(vaccine)	phase I clinical trial	(Schmidt et al., 2012; Yeaman et al., 2014)
	NDV-3A(vaccine)	ongoing clinical trial	(Edwards et al., 2018)
	MAbC7(antibody)	Not determined	(Knight et al., 2005; Brena et al., 2007; Brena et al., 2011)
Hsp90	r-hsp90-CA(vaccine)	Not determined	(Raska et al., 2005)
	pD-HSP90C(vaccine)	Not determined	(Yang et al., 2014)
	SE-CA-*HSP90*(vaccine)	Not determined	(Wang et al., 2006)
	Mycograb	phase III clinical trial	(Matthews et al., 1987; Herbrecht et al., 2006; Pachl et al., 2006; Louie et al., 2011; Bugli et al., 2013)
Eno1	MAb12D9	Not determined	(Ayón-Núñez et al., 2018; Chen et al., 2020)
	CaS1(antibody)	Not determined	(Leu et al., 2010; Leu et al., 2020)
Sap2	PEV-7(vaccine)	phase I clinical trial	(De Bernardis et al., 2012; De Bernardis et al., 2015; Tso et al., 2018)
	hybrid phage displaying Sap2 epitope SLAQVKYTSASSI(vaccine)	Not determined	(Wang et al., 2014)
Hwp1	Hwp1 glycopeptide conjugate(vaccine)	Not determined	(Xin et al., 2008)
	MAb2-E8(antibody)	Not determined	(Coleman et al., 2010; Rosario-Colon et al., 2021; Oh et al., 2022)
Hyr1	recombinant Hyr1 protein	Not determined	(Ghannoum, 2000; Luo et al., 2010; Luo et al., 2011)
Mdh1	recombinant Mdh1p protein	Not determined	(Shibasaki et al., 2014; Shibasaki et al., 2018)

## 2 Agglutinin-like sequence (Als) 3

### 2.1 The structure of Als3

**Figure 2:**
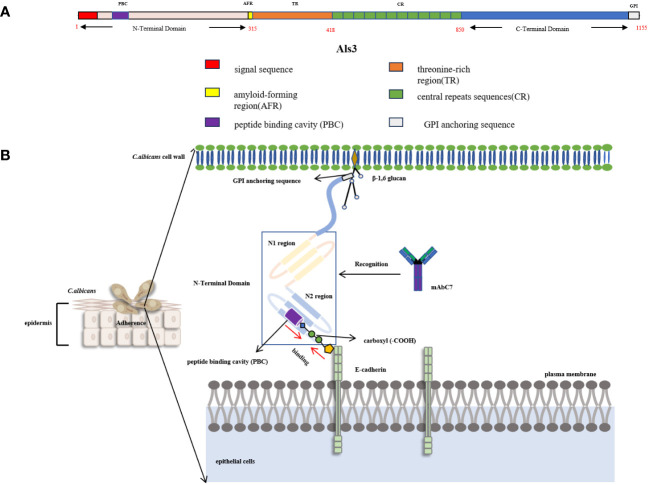
**(A)** Schematic diagram of Als3 structural domain (Hoyer et al., 2008). **(B)** Schematic diagram of the interaction between Als3 and E-cadherin. Als3 is comprised of two consecutive IgG-type immunoglobulin domains (N1, N2) with a peptide binding cavity (PBC) situated in the N2 region. PBC can bind to the carboxyl of E-cadherin in epithelial cells by forming hydrogen bonds. MAbC7 can recognize the N-terminal domain of Als3 and bind to it, thereby blocking the binding between *C.albicans* and host epithelial cells (Zhao et al., 2004; Nobbs et al., 2010; Lin et al., 2014).

The role of Als proteins in the adhesion of *C. albicans* to host cells, a necessary step for host infection, is important. Among the Als family, the fungal cell wall protein Als3 is one of the eight members, functioning as a glycosylphosphatidylinositol (GPI) anchored glycoprotein (Hoyer and Cota, 2016). The N-terminal domain of Als3 initiates with a secretory signal sequence, followed by an amyloid-forming region composed of six amino acids. Following this, it is possible for a conservative sequence abundant in threonine, comprising 103 amino acids, to coexist as a tandem copy with a variable number of repeating sequences, consisting of 36 amino acids (Lin et al., 2014). The quantity of repeat sequences differs among Als proteins, with Als3 harboring 12 such repeats. The C-terminal domain of Als3 concludes with a GPI anchoring sequence, facilitating the attachment of Als3 to the β-1,6-glucan cell wall of *C. albicans* (**Figure 2A**) (Hoyer et al., 2008).

Als3 is a distinctive mycelial-specific protein of *C. albicans* that demonstrates a wide range of substrates for adhesion, including human fibronectin, laminin, collagen, gp96, EGFR (epidermal growth factor receptor), HER2 (human epidermal growth factor receptor-2), N-cadherin, E-cadherin, fibrinogen, casein, and bovine serum albumin (Sheppard et al., 2004). The spatial structure of the N-terminal of Als3(NT-Als3) was examined through X-ray crystal diffraction analysis. Als3 is comprised of two consecutive IgG-type immunoglobulin domains (N1, N2) with a peptide binding cavity (PBC) in the N2 region. In the context of the PBC, the conserved lysine residue (K59) can interact with the ligand’s C-terminal carboxyl group (**Figure 2B**) (Lin et al., 2014). Upon ingress into the PBC, the ligand establishes a hydrogen bond with the neighboring parallel chain (βG2b,^294^TLTWWGY^301^). Concurrently, the water molecular network, characterized by multiple water molecules forming hydrogen bonds between the ligand proteins and PBC, facilitates the interaction between the ligand and the Als3. The variability in the arrangement of the water molecular network enables Als3 to facilitate the impact of *C. albicans* on endothelial and oral epithelial cells, resulting in the adherence of diverse cells and proteins, including fibrin, laminin, and salivary mucosa.

### 2.2 Functions of Als3 in candidiasis

Als3 serves as a structural support in promoting the attachment of *C. albicans* to the host cell surface. More specifically, the C-terminal region of Als3 is anchored to the cell wall by β-1,6-glucan, while its N-terminal domain extends outward to bind with the C-terminal carboxyl group of the host cell surface protein through the PBC domain. The introduction of specific point amino acid mutations (K59M, A116V, Y301F) in Als3 results in the formation of a hydrophobic patch at the terminus of the PBC, leading to a decrease in the hydrophilic interaction between the PBC and the C-terminal of the substrate protein (fibrinogen-γ). Another mutation approach (S170Y) involves the creation of a steric barrier at the PBC terminus, which serves as a “gatekeeper” to impede the entry of peptides at the PBC entrance, thereby preventing the C-terminal of the fibrinogen-γ from accessing the PBC (Lin et al., 2014). The absence of the ALS3 gene has decreased the adherence of *C. albicans* to endothelial and epithelial cells (Zhao et al., 2004; Nobbs et al., 2010). Further studies have demonstrated that the N-terminal domain of recombinant Als3 plays a critical role in facilitating the adhesion of *Staphylococcus aureus* and *C. albicans* in the development of a composite biofilm, highlighting its importance in the adhesion mechanism (Peters et al., 2012).

The interaction between *C. albicans* and epithelial cells can result in endocytosis within the epithelial cells. This process is facilitated by the PBC located in the N-terminal domain of Als3, which binds to the C-terminal carboxyl group of N-cadherin and E-cadherin in the epithelial cells. Als3 further aids in aggregating reticulin and its associated proteins (kinetin, cortisol) from the host at the binding site. This aggregation subsequently leads to the remodeling of actin cytoskeletons and plasma membranes within endothelial cells. Significantly, the deletion of the genes *CDH1* and *CDH2*, responsible for encoding the reticulon protein, resulted in a notable decrease in the endocytosis of endothelial cells in response to *C. albicans* (Moreno-Ruiz et al., 2009). The study demonstrated a significant disparity in mycelial growth between the *als3*Δ/Δ mutant and the wild-type strain, with the former displaying a significantly diminished robustness. Additionally, there was a substantial reduction of nearly 90% in the level of endocytosis by endothelial and epithelial cells (Phan et al., 2005). These findings collectively suggest the indispensability of Als3 in facilitating *C. albicans*’ ability to induce endocytosis.

The role of Als3 in facilitating iron absorption by *C. albicans*, a crucial process for the organism’s survival, has been demonstrated (Weinberg, 1999). In general, the acquisition of iron by biological systems is carefully regulated to prevent the harmful effects of iron overload and the growth impairments caused by iron deficiency (Fourie et al., 2018). Furthermore, hosts, including humans, must restrict iron availability to pathogens. Nevertheless, *C. albicans* can adapt to varying levels of iron, such as the iron-rich conditions in the gastrointestinal tract and the iron deficiency encountered during systemic infection (Fourie et al., 2018). The protein Als3 plays a role in acquiring iron from the host by utilizing a reduction system that extracts iron from ferritin (Almeida et al., 2009). Als3 interacts with host ferritin, leading to the release and uptake of iron through the iron reduction pathway of *C. albicans* (Almeida et al., 2008; Liu and Filler, 2011). However, the specific mechanism by which this occurs is not yet fully understood. When the ALS3 gene of *C. albicans* is expressed in *Saccharomyces cerevisiae*, it enables the binding of ferritin, unlike the closely related members of the Als family, Als1 and Als5 (Almeida et al., 2008). Conversely, the absence of Als3 impairs the growth of *C. albicans* on ferritin plates. Moreover, the absence of transcription factors Tec1 and Bcr1, which play a role in regulating the expression of the *ALS3* gene, reduces iron uptake capability and decreases the mycelial growth and invasive potential of *C. albicans*. Furthermore, it was observed that *C. albicans* hyphae, but not yeast cells, exhibit binding affinity toward ferritin, a critical factor in iron acquisition. This indicates that *C. albicans* can effectively utilize iron from ferritin through morphology-dependent binding facilitated by Als3, thereby highlighting the multifaceted virulence attributes of this singular protein (Lionakis, 2014). Due to its significant role in *C. albicans* adhesion, endocytosis, and iron uptake, Als3 presents itself as a promising candidate for immunotherapeutic interventions aimed at combating candidiasis.

### 2.3 Immunotherapy targeting Als3

The recombinant protein vaccine rAls3p-N, derived from *S. cerevisiae*, encompasses amino acids 17 to 432 in the N-terminal domain of Als3 (Lin et al., 2009). This vaccine acts as an antigen recognized by antigen-presenting cells when combined with immune adjuvants, resulting in the development of CD4^+^ cells by splenic and lymph node lymphocytes. Consequently, the CD4^+^ cells generate Th1/17 cells, which produce IL-17 and IFN-γ, exhibiting strong antifungal effects against *C. albicans*. The administration of this vaccine stimulates the generation of diverse helper T cells, such as Th1, Th17, and Th1/17, thereby facilitating the recruitment of neutrophils and the synthesis of pro-inflammatory factors at the site of infection (Lin et al., 2009). In mice, rAls3p-N can induce cellular immune responses, protecting against invasive *C. albicans* infections (Lin et al., 2008). In the pre-clinical investigation, mice were subjected to subcutaneous injections of a mixture comprising 20 μg of rAls3p-N and Complete Freund’s Adjuvant on day 0. On day 21, the mice were administered another antigen dose with Incomplete Freund’s Adjuvant. After a two-week interval, the mice were intravenously infected with *C. albicans* SC5314 blastospores via the tail vein, and their condition was monitored for 30 days. The findings revealed that the survival rate of mice receiving rAls3p-N injections at a dosage of 20 μg/mice increased to 50%, whereas the control group exhibited a mortality rate of 100%. In the oropharyngeal and vaginal infections model, mice that received rAls3p-N injection exhibited a notable reduction in fungal load compared to those that received adjuvant injection alone (Spellberg et al., 2006).

The vaccine NDV-3, which consists of a tag sequence of rAls3p-N and a 6-His linkage sequence, has exhibited the capacity to induce a robust and safe immune response in individuals who are in good health. This response is characterized by notably elevated levels of anti-Als3 IgG and IgA1 antibodies (Schmidt et al., 2012). Furthermore, NDV-3 has demonstrated its ability to safeguard mice against infections caused by *C. albicans* and methicillin-resistant *S. aureus* by eliciting a potent B and T-cell response (Schmidt et al., 2012; Yeaman et al., 2014). The results of a phase I clinical trial (NCT01273922) indicated that adult participants who were administered a 30 or 300 μg dosage exhibited satisfactory tolerance, generated immune factor IFN-γ, and displayed favorable levels of IL-17A (Schmidt et al., 2012).

Furthermore, an ongoing clinical trial (NCT01926028) is presently examining the effectiveness of an alum formulation of Als3 (NDV-3A, Als3 devoid of extraneous sequences) in the treatment of individuals with a previous record of recurrent vulvovaginal candidiasis. The results of this trial demonstrate that NDV-3A is safe and capable of inducing an immune response. The trial findings have demonstrated that NDV-3A effectively treats vulvovaginal candidiasis, specifically among women under 40. The vaccine has effectively decreased the rates of disease recurrence while simultaneously upholding favorable safety and immunogenicity characteristics (Edwards et al., 2018).

The monoclonal antibody MAbC7 has been found to selectively bind to the N-terminal domain of Als3, thereby hindering the germination and mycelial formation of *C. albicans*. Furthermore, MAbC7 has demonstrated inhibitory properties against the attachment of *C. albicans* to Hep2 cells and buccal epithelial cells, resulting in inhibition rates ranging from 31.1% to 55.3% (Brena et al., 2007). Significantly, MAbC7 effectively targets the iron acquisition pathway of *C. albicans*, impeding the fungal cells’ ability to reduce exogenous iron. Furthermore, this antibody has been observed to enhance the expression of genes linked to iron homeostasis in *C. albicans*, specifically *FET34*, *FTR1*, *FTR2*, and *SIT1* genes (Brena et al., 2011). Fet34 is an essential ferro-oxidase responsible for Fe (II) uptake. The *FTR1* and *FTR2* genes encode membrane iron transporters, while the *SIT1* gene is involved in transporting siderophores for iron uptake. The upregulation of these genes has been shown to decrease the levels of available iron in *C. albicans*, leading to intracellular iron depletion in the fungus (Knight et al., 2005). Nevertheless, the exact mechanism by which MAbC7 binds to the N-terminal of Als3 remains uncertain.

## 3 Heat shock protein 90 (Hsp90)

### 3.1 The structure of Hsp90

**Figure 3:**
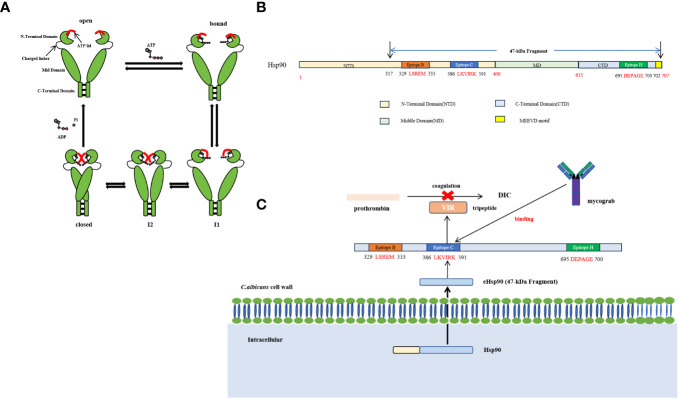
**(A)** The conformational cycle of Hsp90 involves several distinct states. After rapid ATP binding, Hsp90 transitions slowly to the first intermediate state (I1), characterized by open N-domains but a closed ATP lid. Subsequently, the second intermediate state (I2) is formed through N-terminal dimerization, leading to the repositioning of the M-domain and its interaction with the N-domain. Once Hsp90 achieves a fully closed state, ATP hydrolysis occurs. This results in the separation of the N-domains, releasing Pi and ADP, and allowing Hsp90 to return to its open conformation (Shiau et al., 2006; Graf et al., 2009; Biebl and Buchner, 2019). **(B)** Schematic diagram of the recognition epitope and structure of Hsp90 (Meyer et al., 2004; Zierer et al., 2016; Biebl and Buchner, 2019). **(C)** The intracellular fungal Hsp90 can infiltrate and transform into extracellular Hsp90 (eHsp90,47 -kDa Fragment) within the plasma membrane, cell wall, and fungal extracellular space. eHsp90 affects the coagulation pathway as disseminated intravascular coagulation (DIC) is a recognized complication of systemic candidiasis, both eHsp90 and prothrombin carry a tripeptide VIR, which is also located in the LKVIRK epitope of eHsp90 and serves as a catalytic site in prothrombin. Mycograb can recognize and bind to epitope C, thereby blocking the effect of eHsp90 on coagulation processes (Matthews and Burnie, 1992).

Hsp90 is a highly conserved and indispensable molecular chaperone found in eukaryotic cells. Its structural configuration involves a homodimeric form comprising three principal constituents: an N-terminal domain that encompasses a nucleotide-binding pocket and exhibits ATP-binding activity, an ATP-binding region comprising multiple amino acid residues, and characterized by a “lid” structure that assumes a closed conformation in the presence of ATP but an open state in the ADP state, and a Middle domain (M-domain) responsible for facilitating interactions with client proteins and co-chaperones (Ali et al., 2006) (**Figure 3A**). The comparison of functional domains between *C. albicans* and human Hsp90 demonstrates that human Hsp90α share a sequence identity of 71.9%, 68.0%, and 49.2% with *C. albicans* Hsp90 in the N-terminal domain, M-domain, and C-terminal domain, respectively (Li et al., 2021). The M-domain of Hsp90 contains the catalytic arginine essential for ATPase activity and functions as a binding site for co-molecular chaperones, rendering it a crucial site for protein interactions (Meyer et al., 2004). Conversely, the C-terminal domain contributes to the formation of dimers in heat shock proteins (Zierer et al., 2016). Furthermore, the MEEVD motif, comprising the last five amino acids of Hsp90, serves as a binding site for interactions with ubiquitous chaperones with tetrapeptide repeat (TPR) ligands (Biebl and Buchner, 2019). Hsp90 displays restricted ATPase activity and low efficiency in ATP turnover. It is worth noting that the rate of ATP turnover for Hsp90 in yeast cells is once per minute, while in humans, it occurs 10 times per minute (Richter et al., 2001; Richter et al., 2008). Structural analysis findings indicate that Hsp90 exists in a state of dynamic equilibrium, where the binding of nucleotides can initiate a directional conformational cycle (Graf et al., 2009). Hsp90 exhibits rapid ATP binding followed by a gradual transition to the first intermediate state (I1), characterized by open N-domains and a closed ATP lid. The second intermediate state (I2) is formed through N-terminal dimerization, resulting in the repositioning and interaction of the M-domain with the N-domain. Subsequently, Hsp90 achieves a fully closed state, facilitating ATP hydrolysis. Upon hydrolysis, the N-domains dissociate, releasing ADP and inorganic phosphate (Pi), leading to the return of Hsp90 to its open conformation (**Figure 3B**) (Shiau et al., 2006). The protein’s nomenclature is based on its capacity to react to abrupt temperature rises in different cell types, encompassing fungal, animal, and plant cells. Inadequate levels of Hsp90 may result in heightened cell vulnerability to elevated temperatures (Borkovich et al., 1989). Nevertheless, Hsp90’s functional range surpasses its participation in heat shock adaptation, as it also assumes pivotal functions in cell signal transduction, growth, and differentiation. This is accomplished by regulating the conformation of client proteins, facilitating the folding and assembly of newly synthesized proteins, and repairing and degrading albumen damaged by external stimuli (Taipale et al., 2012).

### 3.2 Extracellular Hsp90

Moreover, the intracellular fungal Hsp90 can infiltrate and transform into extracellular Hsp90 (eHsp90) within the plasma membrane, cell wall, and fungal extracellular space (Matthews and Burnie, 1992). In the case of *Aspergillus fumigatus*, eHsp90 is localized within the cell wall and plays a crucial role in preserving cell wall integrity (Lamoth et al., 2012). Additionally, eHsp90 is also present on the surface of *C. neoformans*, and its association with the cell surface depends on the endoplasmic reticulum (ER)-Golgi classical secretory pathway (Chatterjee and Tatu, 2017). Furthermore, the study revealed that the impairment of eHsp90 functionality during and after capsulation resulted in a notable decrease in capsulation size, indicating the potential influence of eHsp90 on the virulence of *C. neoformans* (Chatterjee and Tatu, 2017). It is worth noting that eHsp90 is localized on the cell wall surface and the hyphal tip in *C. albicans* (Burnie et al., 2006). The fungal eHsp90 can also bind to diverse human serum proteins and inhibit their functionality by modulating protein folding or interaction (Matthews and Burnie, 1992). In the investigation of *C. albicans*, an immunodominant 47-kDa antigen originating from the C-terminal of Hsp90, which exhibited heat stability, was discovered (Matthews and Burnie, 1989). Initially, this 47-kDa antigen was acknowledged as a noteworthy focal point of the immune response in individuals afflicted with invasive candidiasis, and subsequent research has demonstrated its association with survival rates in both human and animal models of infection (Matthews and Burnie, 1988; Matthews and Burnie, 1989). The presence of antibodies to the 47-kDa antigen has been observed in the majority of patients with chronic mucocutaneous candidiasis and acquired immune deficiency syndrome (Burford-Mason et al., 1987). Furthermore, eHsp90 demonstrates exogenous biological activities within the abnormal extracellular environment. Notably, it has the potential to impact the coagulation pathway, as disseminated intravascular coagulation (DIC) is a well-established complication of systemic candidiasis. Both eHsp90 and prothrombin possess a tripeptide VIR, which is also situated within the LKVIRK epitope of eHsp90 and functions as a catalytic site in prothrombin (Matthews and Burnie, 1992) (**Figure 3C**). These all suggest its potential role in mediating the protective effect of humoral immunity in invasive candidiasis (Matthews et al., 1988; Matthews et al., 1991). Epitope mapping showed that patients recovering from invasive candidiasis produce antibodies against fungal Hsp90 by binding epitope C (^386^LKVIRK^391^) as the most prevalent, followed by epitope B (^329^LSERM^333^) as the second most commonly recognized and epitope H (^695^DEPAGE^700^), exclusively specific to *C. albicans* and located at the C-terminal, as the third commonly recognized epitope observed in approximately 50% of the subjects (Matthews, 1992).

### 3.3 Immunotherapy targeting Hsp90

Due to the significant contribution of eHsp90 in the progression of candidiasis, there has been substantial interest in developing vaccines and antibodies that target Hsp90. The exploration of Hsp90 antibodies can be traced back to the 1980s when Matthews et al. discovered a positive association between the levels of anti-Hsp90 antibodies and patient prognosis and mortality (Matthews et al., 1987). Mycograb, a 28-kDa recombinant human antibody fragment, encompasses the antigen binding domain of both the heavy and light antibody chains and exhibits specific binding to an epitope, ^386^LKVIRK^391^, in fungal Hsp90 (Herbrecht et al., 2006). The antifungal effects of Mycograb were synergistic when combined with amphotericin B and caspofungin. In a mouse model of systemic candidiasis with a normal immune response, Mycograb exhibited superior efficacy in killing *C. albicans* compared to treatment with amphotericin B alone. Furthermore, a multinational phase III clinical trial demonstrated that combining Mycograb and amphotericin B lipid-related preparations significantly reduced the mortality rate associated with *Candida* species in patients with invasive candidiasis, surpassing the efficacy of amphotericin B monotherapy. The combination therapy demonstrated a notable enhancement in efficacy, as evidenced by the increase in survival rate from 48% in amphotericin B monotherapy to 84% in combination therapy. This improvement in patient outcomes was further supported by the notable increase in culture-confirmed *C. albicans* clearance rate (Pachl et al., 2006). However, the Mycograb binding antigen epitope (^386^LKVIRK^391^) represents a relatively conserved segment within both human and fungal Hsp90. Regrettably, this characteristic also enables Mycograb to interact with the host’s Hsp90, thereby inducing a potentially severe cytokine release syndrome. As a result, the Committee for Medicinal Products for Human Use rendered an unfavorable judgment in November 2006, advising against the approval of Mycograb for marketing due to apprehensions regarding its quality and safety (Bugli et al., 2013). Considering this decision, Arnold Louie and colleagues endeavored to tackle the challenges arising from the disparities in molecular weight and conformational structure of Mycograb (Louie et al., 2011; Bugli et al., 2013). Consequently, they devised a revised version of Mycograb, known as the Mycograb C28Y variant, by substituting cysteine at position 28 with tyrosine. However, the co-administration of amphotericin B and doses up to 15 mg/kg per day of the Mycograb C28Y variant did not exhibit any enhanced efficacy compared to amphotericin B monotherapy in a murine model of invasive candidiasis under conditions of neutropenia (Louie et al., 2011).

Furthermore, some vaccines have been specifically formulated to target Hsp90. Raska et al. conducted a study in which they created a DNA vaccine expressing Hsp90 of *C. albicans* and a recombinant protein vaccine (r-hsp90-CA) to evaluate their effectiveness in combating candidiasis (Raska et al., 2005). The findings suggest that the levels of hsp90-specific IgG generated through DNA vaccination were approximately one-third of those produced through protein vaccination. The difference in effectiveness can be ascribed to the comparatively limited expression of the Hsp90 antigen from DNA vaccines following injection (Raska et al., 2005). Consequently, subsequent investigations have employed the concurrent administration of an immunologic adjuvant and DNA vaccine to augment the immunogenicity of the DNA vaccine. Wang et al. utilized polysaccharides derived from the fruits of *Physalis alkekengi* L as the immunologic adjuvant in conjunction with the DNA vaccine. The DNA vaccine pD-HSP90C consists of a recombinant plasmid containing epitope C (^386^LKVIRK^391^) of Hsp90 from *C. albicans* and polysaccharides. The results show that the concurrent administration of polysaccharide and DNA vaccine significantly enhances mice’s humoral and cellular (Th1, Th2) immune responses while substantially extending their survival time against systemic candidiasis (Yang et al., 2014).

Additionally, a study successfully engineered a vaccine that expressed a specific epitope H (^696^DEPAGE^700^) of *C. albicans* Hsp90(SE-CA-*HSP90*) on the surface of filamentous phage, fused to the major coat protein pVIII. The protective immune responses of the hybrid-phage vaccine were investigated in mice. In the survival rate experiment, the data showed that 60% of the mice immunized with the hybrid-phage vaccine survived after 15 days, whereas only 11% of the mice immunized with TE (Tris-EDTA) and 20% of the mice immunized with wild-type phage were observed to be alive on the 15 th day after infection. Furthermore, the presence of the SE-CA-HSP90-specific antibody was exclusively detected in the mice that received the hybrid-phage vaccine. Moreover, the delayed-type hypersensitivity (DTH) response and natural killer cell activity exhibited significant augmentation compared to the TE control group (Wang et al., 2006).

## 4 Eno1

### 4.1 The structure of Eno1

Enolase plays a crucial role in the glycolysis pathway by catalyzing the conversion of 2-phosphoglycerate to phosphoenolpyruvic acid (Kang et al., 2008). In *C. albicans*, the Eno1 protein comprises 440 amino acids and has a molecular weight of approximately 47 kDa. Eno1 is abundant in *C. albicans*, accounting for 0.7% and 2% of the total protein content during the yeast and mycelium phases (Sundstrom and Aliaga, 1994). The three-dimensional structure of Eno1 was determined through X-ray diffraction analysis of the complex formed by Eno1 and 2-phosphoglycerate. The diffraction data unveiled that four Eno1 proteins assemble into two dimers within an asymmetric unit, interconnected by four pairs of hydrogen bonds. Two Eno1 molecules form homologous dimers, exhibiting an antiparallel orientation (Li et al., 2022).

### 4.2 Functions of Eno1 in candidiasis

Eno1, a protein widely distributed in the interior and surface of *C. albicans*, possesses multifunctional properties (Gil-Bona et al., 2018). Specifically, Eno1 primarily functions in glycolysis and gluconeogenesis, exhibiting enolase activity in the cytoplasm of *C. albicans*. The knockout of the *ENO1* gene results in the inability of *C. albicans* to survive on media containing glucose as the sole carbon source (Ko et al., 2013). Despite the absence of a conventional secretory signal, the surface exposure of Eno1 relies on the presence of the first 28 amino acids in the N-terminal domain and a Tlg2-dependent pathway in *S. cerevisiae* (Miura et al., 2012). Moreover, it has been suggested that the N-terminal domain of Eno1, consisting of 169 amino acids, may have a role in sorting processes; alternatively, Eno1 could be transported via extracellular vesicles (López-Villar et al., 2006; Karkowska-Kuleta et al., 2020). Eno1 has been found to possess transglutaminase activity on the cell wall of *C. albicans* and is classified as a type of cell wall integrin (Ruiz-Herrera et al., 1995). Transglutaminase functions by catalyzing N- ϵ-(γ- glutamyl lysine amide) bonds at its active center, resulting in covalent cross-linking within or between proteins (Ruiz-Herrera et al., 1995). A substrate for transglutaminase, hyphal wall protein 1 (Hwp1) is exclusively expressed during the hyphal state of *C. albicans* and is characterized by its high content of glutamine residues. The serine/threonine enrichment site of Hwp1 undergoes glycosylation and is subsequently secreted by a signal peptide, ultimately anchoring to the cell wall surface through GPI (Staab et al., 2004). Eno1 enzymatically interacts with Hwp1 and catalyzes covalent cross-linking between the glutamine residue of Hwp1 and the lysine residue of the N-cadherin and E-cadherin of epithelial cells, causing *C. albicans* to adhere to the host cell and promoting infection (Staab et al., 1999).

Extracellular Eno1 exhibits binding affinity toward various molecules, including cadherin in endothelial cells, laminin in the basal membrane, and fibronectin and vitronectin in the extracellular matrix. This binding capability contributes to the pathogenicity of fungi by disrupting the host’s homeostasis/hemostasis system through the initiation of protein hydrolysis cascades, such as fibrinolysis, complement activation, and the kallikrein-kinin system (Felk et al., 2002; Wächtler et al., 2012; Silva et al., 2014; Kozik et al., 2015; Satala et al., 2020). Additionally, Eno1 is capable of binding to human plasminogen, which is typically found on the surface of mammalian cells and within the fibrin network. This interaction is crucial in fibrin and extracellular matrix breakdown during tissue remodeling (Keragala and Medcalf, 2021). The primary binding sites for human plasminogen on Eno1 are the two C-terminal lysine residues (Derbise et al., 2004). Furthermore, employing cross-linking analysis, it has been determined that the sequence ^237^AGYKGKVGIAMDVASSEFYKDGK^259^ is a significant elongated fragment essential for the interaction between Eno1 and human plasminogen (Satala et al., 2020).

### 4.3 Immunotherapy targeting Eno1

The monoclonal antibody underwent screening by injecting recombinant Eno1 into mice, producing monoclonal antibodies. The findings demonstrated that 12D9 displayed selective binding properties to Eno1 in the cell wall of *C. albicans*, effectively impeding its interaction with plasmin. Prior research has indicated that the 251-259 position within the six-helix six sheets 6 (H6S6) loop of the EnoA structure of *Taenia solium* is the binding motif for plasmin (Ayón-Núñez et al., 2018). A comparative analysis involving multiple *Candida* species showed that the sequence composition exhibited a notably high similarity. Subsequently, it was ascertained that the 12D9 antibody specifically targeted the ^254^FYKDGKYDL^262^ motif within the Eno1 H6S6 protein, impeding its interaction with plasmin and subsequently inducing plasmin activation. The *in vitro* experimental results proved that 12D9 effectively alleviated the adverse impacts of *C. albicans* on human umbilical vein endothelial and Caco-2 cells. The administration of 12D9 in a mouse model resulted in a notable increase in the survival rate of mice and a reduction in the fungal load within their kidneys. Moreover, the combination of 12D9 with fluconazole and anidulafungin demonstrated a synergistic antifungal effect (Chen et al., 2020).

By subjecting the antibodies generated during the seventh humoral immunity process in chickens to screening and modification, researchers successfully isolated a single-chain variable antibody known as CaS1 (Leu et al., 2010). *In vitro* experiments revealed that the antifungal potency of scFv CaS1, at a concentration of 150 ug/mL, is comparable to that of fluconazole, effectively inhibiting the growth of *C. albicans*. Furthermore, by binding this antibody with various species of *Candida* Eno1, it was observed that only Eno1 derived from *C. albicans* and *Candida tropicalis* exhibited specific recognition by CaS1 (Leu et al., 2020). *In vivo* animal model experiments were conducted to investigate the effects of CaS1 on *C. albicans* adhesion ratio in zebrafish. The results showed that after exposure to CaS1 for 2 and 4 hours, there was a significant decrease of 21% and 35% in the adhesion ratio, respectively. Furthermore, the administration of CaS1 in mice resulted in a notable reduction in fungal burden and associated cytokine levels (Leu et al., 2020).

## 5 Secretory aspartate protease (Sap)

### 5.1 The structure of Sap

The secretion of Sap by *C. albicans* hyphae, an acid protease characterized by aspartic acid residues at its active site, aids in the infiltration of host cells (Mandujano-González et al., 2016). *SAP* genes have been observed in multiple pathogenic *Candida* species, such as *C. albicans*, *C. dubliniensis*, *C. tropicalis*, and *C. parapsilosis* (de Viragh et al., 1993; Gilfillan et al., 1998; Zaugg et al., 2001). Research has shown that *SAP* genes display unique regulatory patterns and serve specific functions at different stages of infection, likely due to the diversity of coding gene types and variations among *Candida* species (Schaller et al., 2001). The expressions of *SAP1*, *SAP2*, and *SAP3* genes have been exclusively observed in the yeast form of *C. albicans* among the 10 SAP genes. Sap2 has been identified as a crucial contributor, particularly necessary for *in vitro* growth, specifically when bovine serum albumin is the sole carbon source (White et al., 1993). In contrast, the *SAP4*, *SAP5*, and *SAP6* genes are exclusively expressed in the mycelial form and are associated with invasive candidiasis and evasion of the host immune system (Borg-von Zepelin et al., 1998). The presence of *SAP7* has been observed in mouse models, but it has not been observed in any *in vitro* settings. Its expression is associated with the virulence of venous infections (Taylor et al., 2005). Moreover, previous research has demonstrated that *SAP8* is rapidly expressed in yeast and epithelial models (Naglik et al., 2008). Additionally, the proteins encoded by the *SAP9* and *SAP10* genes contain GPI-anchored domains, indicating their potential role in maintaining cell wall integrity (Albrecht et al., 2006).

The synthesis of Sap initiates in the nucleus, where the newly formed mRNA is subsequently transported to the cytoplasm for translation into a proenzyme that exceeds the mature protease by 60 amino acids. In the rough endoplasmic reticulum, the N-terminal signal peptide is cleaved by the signal peptide enzyme, and the resulting zymogen is then transported to the Golgi apparatus. Within the Golgi apparatus, the zymogen undergoes additional processing facilitated by Kex2 protease, a Ca^2+^-dependent serine protease belonging to the subtilisin-like proprotein convertase family (Brenner and Fuller, 1992), and it has high specificity for the cleavage of C-terminal paired basic sites, most often Lys-Arg, Lys-Lys or Arg-Arg following the Lys Arg sequence (von Heijne, 1985; Togni et al., 1996). The mature form of Sap, a protein produced by *C. albicans*, displays a size range of 35 to 50 kDa (Monod et al., 1998). Saps possess distinctive sequence motifs, including two conserved aspartic acid residues at the active site and conserved cysteine residues that contribute to maintaining their three-dimensional structure (Naglik et al., 2003). Sap2 is the most abundant among the Saps when proteins are the sole nitrogen source (Naglik et al., 2003). Sap2 exhibits typical characteristics of Saps, featuring a monomeric structure composed of N- and C-terminal domains, which contain an active site region. The structural configuration of Sap2 is reminiscent of a bilobed structure characterized by a central fissure that can accommodate peptide substrates containing up to nine residues (Calugi et al., 2013). The N-terminal domain of Sap2 displays an Asp-Thr-Gly motif, while its C-terminal domain exhibits an Asp-Ser-Gly motif. In contrast, most mammalian aspartate proteases feature an Asp-Thr-Gly motif in both domains (Cutfield et al., 1995; Koelsch et al., 2000).

### 5.2 Functions of Saps in candidiasis

Saps play a pivotal role in various processes of *C. albicans*, including adhesion, hyphal growth, biofilm formation, and immune evasion. An Arg-Gly-Asp motif enables Sap 4, Sap5, and Sap6 to bind to an integrin receptor on the epithelial cell surface, facilitating the adhesion of *C. albicans* to host cells (Wu et al., 2013). An oral mouse model of candidiasis was utilized to evaluate the *in vivo* adhesion function of Sap6. The adhesion rate of wild-type *C. albicans* cells was 78 ± 1%, whereas the *sap6*Δ/Δ mutant exhibited a significant reduction of 60% in adhesion compared to the wild-type strain (Kumar et al., 2015).

Sap9 governs the growth of hyphae and the formation of biofilms in *C. albicans* utilizing a cAMP-dependent pathway that encompasses a newly identified G protein-coupled receptor, Gprl, as well as the G proteins Gpa2 and Ras2, adenylyl cyclase (AC), cyclic AMP (cAMP), and cAMP-dependent protein kinase (PKA) (Midkiff et al., 2011; Yang et al., 2020). Activation of this pathway occurs in response to environmental nutrients, such as glucose and amino acids, which are detected by the G protein-coupled receptor (GPCR) located on the cell surface. Guanine nucleotide-binding protein (G protein) activation occurs through signal detection, increasing intracellular cAMP levels during adenylate cyclase stimulation. This elevation of cAMP subsequently triggers the activation of PKA, which in turn phosphorylates downstream target proteins. In fungal pathogens, these biochemical reactions are associated with morphological alterations and the release of virulence factors (Leberer et al., 2001; Liebmann et al., 2004). Notably, studies have demonstrated the significant involvement of Sap9 in the initiation and sustenance of hyphal development (Yang et al., 2020). The precise mechanism by which Sap regulates hyphal morphogenesis remains uncertain; however, it is hypothesized that Sap9 may exert a proteolytic influence on Efg1, thereby facilitating its activation. In order to validate the impact of Sap9 on the hyphal induction pathway, this investigation documented a significant decrease of approximately 40% in Efg1 expression within strains lacking Sap9 when subjected to a hyphal induction medium. These findings suggest that Sap9 governs hyphal formation by modulating Efg1 expression via the cAMP-dependent pathway (Yang et al., 2020).

### 5.3 Immunotherapy targeting Sap2

The vaccine candidate Sap2, derived from *C. albicans*, has been the subject of extensive research due to its involvement in fungal virulence (Naglik et al., 2003). The *SAP2* gene of *C. albicans* plays a significant role in fungal pathogenesis by degrading host proteins at epithelial sites and hydrolyzing complements (Naglik et al., 2003). De Bernardis et al. conducted a study demonstrating that *Candida* mutant strains lacking Sap2 displayed reduced virulence in a rat model of vaginal candidiasis compared to strains with Sap1 to Sap6 intact (De Bernardis et al., 1999). The immunization of mice using recombinant Sap2 has shown to be an effective method of protecting against invasive candidiasis. Additionally, the anti-Sap2 antibody (IgG) transfer has significantly decreased *C. albicans* infection (von Heijne, 1985). Classical immunization with Sap2 and alum as an adjuvant has resulted in a strong humoral response, substantially reducing *C. albicans* burden in the kidney during infection compared to non-immunized animals (Vilanova et al., 2004). Moreover, in a mucosal model, mice intranasally immunized with Sap2 demonstrated reduced fungal burdens following oral and vaginal challenges with *C. albicans* (Rahman et al., 2012). In conjunction with antibodies, several vaccines targeting Sap2 have exhibited effectiveness. An example of such a vaccine is PEV-7, which consists of a genetically engineered truncated form of Sap2 lacking 20 amino acids from the N-terminal region of the fully mature Sap2. This truncated Sap2 variant was integrated into influenza virosomes. Rats immunized with PEV-7 through the intravaginal pathway demonstrated safeguarding against vaginitis induced by *C. albicans* through antibodies (De Bernardis et al., 2012). In addition, the PEV-7 virosomal vaccine formulation has completed a Phase I clinical trial (NCT01067131), demonstrating that the administration of the PEV7 vaccine through intramuscular injection or intravaginal capsules induced a strong B cell-mediated immune response in vaginal and cervical samples (De Bernardis et al., 2015; Tso et al., 2018). Moreover, BALB/c mice vaccinated with either the hybrid phage displaying the Sap2 epitope ^382^SLAQVKYTSASSI^393^ or the recombinant Sap2 displayed vigorous cellular and protective humoral responses against *C. albicans* infection. Significantly, the findings of this study demonstrated that the hybrid phage immunization approach resulted in levels of protection that were comparable to those achieved through recombinant protein immunization. Specifically, both the group immunized with hybrid phage and the group immunized with recombinant Sap2 exhibited a significant reduction in the number of *C. albicans* cells at day 7 following the initial challenge. Furthermore, upon completion of the second survival challenge, mice were inoculated with 10^7^ C*. albicans* cells. It became evident that the administration of hybrid phage or the recombinant Sap2 elicited an immune response and provided safeguarding against a fatal challenge in a mouse model (Wang et al., 2014).

## 6 Hyphal wall protein 1(Hwp1)

### 6.1 The structure of Hwp1

The adhesin protein Hwp1, frequently found on the surfaces of germ tubes and mycelium in *Candida* species, serves as a substrate for host cell transglutaminase. This enables covalent binding and cross-linking between the *Candida* genus and mucosal epithelial cells (Chaffin, 2008; Romeo and Criseo, 2008). The Hwp1 adhesin has also been proposed to contribute to the retention of *C. albicans* within biofilms *in vivo* (Mirhendi et al., 2011).

The *HWP1* gene, responsible for encoding a protein featuring a surface-exposed N-terminal domain of Hwp1 that can bind to external ligands like antibodies, was discovered via immune screening of the germ tube cDNA library. Like Als3, Hwp1 is affixed to the cell wall through GPI through its C-terminal (Staab et al., 1996). Furthermore, the cDNA-derived amino acid sequence showcases a succession of 10 amino acid tandem repeats, constituting most of the open reading frame. The molar percentage of amino acids in the open reading frame indicates that proline accounts for 27%, glutamine accounts for 16%, and aspartic acid accounts for 12% of the composition. The C-terminal region of Hwp1 exhibits a high abundance of threonine and serine, providing numerous potential sites for O-glycosylation (Staab et al., 1996). The significant presence of proline residues and the repetitive sequence length(14 X 10 AA tandem repeats of [EVIQ]-P-[CDT]-D-[YNW]-P-[PQ]-[QI]-[QP]-[QDN]) classify Hwp1 within various proline-rich proteomes, where proline residues are recognized for their ability to maintain the extended conformation of peptide chains and facilitate non-covalent interactions between protein chains or in the case of salivary proteins, binding to toxic plant polyphenols (Williamson, 1994). The recurrence of cysteine residues potentially aids in generating extracellular disulfide bonds at consistent intervals, consequently facilitating the cross-linking of Hwp1 and other protein molecules on the cell surface (Chattaway et al., 1974). Additionally, including acidic amino acids in the repetitive sequence potentially imparts negative charges to the mycelium surface under physiological pH conditions (Holmes et al., 1992).

### 6.2 Functions of Hwp1 in candidiasis

The presence of Hwp1 is crucial for forming biofilms by *C. albicans*, both *in vivo* and *in vitro*. This protein is situated on the cellular surface and is connected to cell wall glucan through the remaining portion of its GPI anchor (Staab et al., 2004). *In vitro* experiments have shown that a *C. albicans* mutant lacking the *HWP1* gene exhibits diminished biomass in biofilm production compared to the wild-type strain (Nobile et al., 2006a). Additionally, the *hwp1*Δ/Δ mutant produces a biofilm with a depth of approximately 100 µm that contains few hyphae. In contrast, reconstitution with a wild-type *HWP1* allele permits the production of a biofilm with a depth of 200 to 300 µm, in which hyphae are readily present. The experimental findings suggest that Hwp1 plays a crucial role in the *in vitro* production of biofilms. Furthermore, a comparison of biofilm formation between *hwp1*Δ/Δ mutant and wild strains in a rat venous catheter model revealed notable distinctions that the *hwp1*Δ/Δ mutant had a severe biofilm defect: only sparse microcolonies were observed on the catheter, and the microcolonies were devoid of hyphae (Nobile et al., 2006b).

### 6.3 Immunotherapy targeting Hwp1

Recently, Hoyer and co-workers developed a monoclonal antibody against Hwp1 of *C. albicans*. The Gln-Pro-rich adhesive domain (CDNPPQPDQPDDN (amino acids 154 to 166 of the protein)) of Hwp1 was utilized to produce monoclonal antibody MAb 2-E8 (Oh et al., 2022). The result showed that 2-E8 can bind to Hwp1 specifically. MAb 2-E8 labeled the germ tube of the wild-type strain but not *the hwp1*Δ/Δ strain (Oh et al., 2022). Furthermore, assays were performed to assess the blocking potential of MAb2-E8 on adhesive interactions. As a control, Anti-Als1 MAb1-B2, which was previously shown to inhibit *C. albicans* adhesion to biliary epithelial cells (BECs), was included (Coleman et al., 2010). Results indicated that MAb2-E8 at a 20 µg/mL concentration effectively blocked adhesion, like Anti-Als1 (Oh et al., 2022). Furthermore, Rosario-Colon et al. found that *Candida* Hwp1-specific monoclonal antibodies 6H1 could protect mice against invasive *C. auris* infection by significantly enhancing survival and reducing fungal burdens (Rosario-Colon et al., 2021). Moreover, vaccination with glycopeptide conjugate consisting of β-mannan polysaccharide combined with Hwp1 peptide epitopes (QGETEEALIQKRSY) showed protection against experimental disseminated candidiasis in mice by favoring the production of protective and specific antibodies (Xin et al., 2008).

## 7 Other potential targets

In addition to the proteins mentioned above, numerous other proteins are also implicated in the pathogenesis of candidiasis. One such protein is hyphal-regulated protein 1 (Hyr1), which is crucial for hyphal growth and contributes to the virulence of *C. albicans* by evading phagocyte killing, a key host defense mechanism against candidiasis (Luo et al., 2010). Previous investigations have demonstrated that both active and passive immunity conferred by a recombinant N-terminal fragment of Hyr1 confer protection against lethal candidemia in mice (Luo et al., 2011). Consequently, it is postulated that vaccination with Hyr1 may exert a protective effect against candidiasis. Furthermore, it has been identified that phospholipase B serves as a virulence factor in *C. albicans* (Ghannoum, 2000). Moreover, the serum of individuals afflicted with invasive candidiasis exhibits the presence of antibodies that exhibit reactivity toward purified phospholipase B, thereby rendering it a compelling candidate for diagnostic and antibody preparation applications (Heilmann et al., 2011). Additionally, the indispensable role of malate dehydrogenase (Mdh1) in the tricarboxylic acid cycle, coupled with its involvement in aerobic energy production via participation in the malate-aspartate shuttle, has been established (Shibasaki et al., 2018). Mdh1 has been identified as a potential vaccine antigen for candidiasis due to its consistent presence throughout the investigated time points and lack of significant variation in relative abundance (Shibasaki et al., 2018). Investigations into the use of recombinant Mdh1 as a candidate antigen for a candidiasis vaccine have demonstrated that subcutaneous and intradermal administration in mice elicits significantly elevated antibody responses and confers significant protection against candidiasis (Shibasaki et al., 2014).

## 8 Conclusions and future perspectives

*C. albicans*, a pathogen characterized by its opportunistic nature, poses an increasing threat to human health. The proteins adhere to and invade host cells in symbiotic and pathogenic scenarios involving *C. albicans.* The morphological changes and accompanying physiological processes exhibited by this pathogen serve various purposes and offer promising avenues for drug investigation. Antibody-based medications targeting these proteins have exhibited positive therapeutic outcomes. In addition, antifungal antibodies present a diminished occurrence of adverse reactions and a broader array of choices when compared to small molecule drugs. Furthermore, the utilization of a synergistic combination of monoclonal antibodies and antifungal medications not only provides a more comprehensive and efficient approach to combat the escalating drug resistance observed in *Candida* species but also enhances specificity and holds potential for improved clinical outcomes. Additionally, the advancement of novel immunomodulatory techniques that integrate the regulation of recombinant cytokines with monoclonal antibodies presents an alternative strategy to enhance the therapeutic efficacy of the latter. Moreover, emerging technologies offer promising avenues for the treatment of life-threatening invasive fungal infections. However, despite the encouraging potential for vaccine development and antifungal therapies, additional research is necessary to comprehend their effectiveness and constraints comprehensively. It is crucial to explore approaches for establishing a persistent and uniform symbiotic model of candidiasis in mice, considering its symbiotic nature in humans. Understanding the mechanisms contributing to the effectiveness of relevant vaccines and antibodies is paramount to appropriately administering them to eligible patients and maximizing their therapeutic benefits. Additionally, it is imperative to establish a timely and dependable method for evaluating the efficacy of vaccines and antibodies while also investigating the potential of antibodies as valuable biomarkers for diagnosing *Candida* infection. Moreover, the primary challenge lies in securing adequate funding for clinical trials of innovative antifungal medications, particularly for vaccinations targeting opportunistic fungal pathogens that exclusively affect individuals with severe immune system deficiencies. These vaccinations may not be as commercially appealing as those designed to combat diseases that affect a significant portion of the general population. Consequently, it is anticipated that collaborative efforts involving academia, industry, and the government will be imperative in facilitating the advancement of promising and novel antifungal immunotherapeutics into clinical trials and, subsequently, the market.

## Author contributions

ZF: Data curation, Formal analysis, Visualization, Writing – original draft. HL: Conceptualization, Supervision, Writing – review & editing. YJ: Conceptualization, Funding acquisition, Supervision, Writing – review & editing.
